# Leveraging Knowledge Graphs and Natural Language Processing for Automated Web Resource Labeling and Knowledge Mobilization in Neurodevelopmental Disorders: Development and Usability Study

**DOI:** 10.2196/45268

**Published:** 2023-04-17

**Authors:** Jeremy Costello, Manpreet Kaur, Marek Z Reformat, Francois V Bolduc

**Affiliations:** 1 Department of Electrical and Computer Engineering University of Alberta Edmonton, AB Canada; 2 Department of Pediatrics University of Alberta Edmonton, AB Canada; 3 Information Technology Institute University of Social Sciences Łódź Poland; 4 Department of Medical Genetics University of Alberta Edmonton, AB Canada; 5 Women and Children Health Research Institute University of Alberta Edmonton, AB Canada; 6 Neuroscience and Mental Health Research Institute University of Alberta Edmonton, AB Canada

**Keywords:** knowledge graph, natural language processing, neurodevelopmental disorders, autism spectrum disorder, intellectual disability, attention deficit hyperactivity disorder, named entity recognition, topic modeling, aggregation operator

## Abstract

**Background:**

Patients and families need to be provided with trusted information more than ever with the abundance of online information. Several organizations aim to build databases that can be searched based on the needs of target groups. One such group is individuals with neurodevelopmental disorders (NDDs) and their families. NDDs affect up to 18% of the population and have major social and economic impacts. The current limitations in communicating information for individuals with NDDs include the absence of shared terminology and the lack of efficient labeling processes for web resources. Because of these limitations, health professionals, support groups, and families are unable to share, combine, and access resources.

**Objective:**

We aimed to develop a natural language–based pipeline to label resources by leveraging standard and free-text vocabularies obtained through text analysis, and then represent those resources as a weighted knowledge graph.

**Methods:**

Using a combination of experts and service/organization databases, we created a data set of web resources for NDDs. Text from these websites was scraped and collected into a corpus of textual data on NDDs. This corpus was used to construct a knowledge graph suitable for use by both experts and nonexperts. Named entity recognition, topic modeling, document classification, and location detection were used to extract knowledge from the corpus.

**Results:**

We developed a resource annotation pipeline using diverse natural language processing algorithms to annotate web resources and stored them in a structured knowledge graph. The graph contained 78,181 annotations obtained from the combination of standard terminologies and a free-text vocabulary obtained using topic modeling. An application of the constructed knowledge graph is a resource search interface using the ordered weighted averaging operator to rank resources based on a user query.

**Conclusions:**

We developed an automated labeling pipeline for web resources on NDDs. This work showcases how artificial intelligence–based methods, such as natural language processing and knowledge graphs for information representation, can enhance knowledge extraction and mobilization, and could be used in other fields of medicine.

## Introduction

Access to curated medical information has become more important than ever due to the growing amount of information available on the internet and the many challenges faced with sharing information about medical topics. Neurodevelopmental disorders (NDDs) are a range of conditions, including autism spectrum disorder, intellectual disability, and attention deficit hyperactivity disorder. These disorders affect up to 18% of the population [1-7] and are influenced by the growing amount of online information and misinformation [8,9]. NDDs have complex medical features, and the needs of affected individuals and their families tend to be quite diverse [10-12].

There exists a large amount of information relating to NDDs on the internet, but this information is scattered across many websites, often using different terminology and containing both reliable information and misinformation. Finding information that is specific, relevant, and trusted is therefore difficult for the caregivers of children with NDDs. To remedy this, a knowledge repository containing available NDD resources annotated with appropriate labels and terms could be constructed. This repository would enable the discovery of relevant trusted resources based on phrases of interest provided by users.

We propose the use of a knowledge graph (KG) to represent web resources together with terms and phrases annotating them. The use of a KG enables web links (ie, resources), terms, and phrases to be represented as nodes, with the relevance between them represented as edges.

A KG indexing web links and information on NDDs would allow experts and nonexperts to have a primary repository of NDD knowledge. With this knowledge, doctors could make quicker and more accurate selections of relevant resources/websites, and caregivers of children with NDDs could quickly find appropriate information, services, and financial support. Accurate identification and early help are critical to quality of life outcomes for those with NDDs. The proposed graph-based repository could improve many peoples’ lives.

This paper describes the methodology of constructing a KG-based repository of NDD resources. It presents the following:

- An approach for automatic processing of text extracted from websites relating to NDDs and identification of the most accurate terms/phrases describing them based on named entity recognition (NER), topic modeling, location detection, and resource classification.
- A process of determining degrees of relevance between KG entities and resources, and storing them as weights of relations in the graph.
- An application of the ordered weighted averaging (OWA) operator [13] for determining the most relevant resources using the aggregated weights of relations between resources and terms/phrases describing them.
- An example of using the constructed KG-based repository of NDD resources for retrieving a ranking of resources related to a phrase representing the user’s interests.

The paper reviews some related works and describes the methodology used for constructing a KG. It also includes an overview of the KG schema, gives an in-depth look at individual techniques used to annotate scraped web resources, and introduces an aggregation process. Finally, a brief overview of the use of the constructed graph is presented, and an outline of the conclusion and possible future work is provided. KGs have been used in many areas, including medicine, cyber security, finance, news, and education. There have been a wide range of KG applications within the medical field. Applications include general KGs across the whole medical domain and across specific areas, such as depression, thyroid disease, and COVID-19, as described below.

Several KGs spanning the entire medical field have been created. For example, Ernst et al [14] created KnowLife. They used advanced information extraction methods, including NER, pattern mining, and consistency reasoning, to populate entities and relations from scientific literature and online communities, in contrast to many previous works involving manual curation. Shi et al [15] developed methods to extract syntactic, semantic, and structural information from conceptual KGs. They used a similar method to KnowLife for creating the KGs, and extended understanding of the resultant KGs by using machine learning methods to prune meaningless relations in the graphs and extract semantic knowledge. Sheng et al [16] created DEKGB, a KG of various diseases, using prior medical knowledge and electronic medical records, along with guidance from doctors. Li et al [17] used quadruplets instead of triples to represent their KG, with extra information relating to relation strength. Zhang et al [18] used a clinician-in-the-loop approach to fine-tune an automated KG construction method. KGs have also been created for specific medical domains. Huang et al [19] made a KG solely focused on depression after observing the prohibitive size and high-level nature of general medical KGs. A low-level KG for depression would allow more convenient use by doctors, easier understandability by the public, and higher computational efficiency. Chai [20] used a KG about thyroid disease as the backbone for an intelligent medical diagnosis system. Vector embeddings were calculated for each entity and relation in the KG. These embeddings were then used to train a bidirectional long short-term memory (LSTM) network as a disease diagnosis model, outperforming other tested machine learning models. Flocco et al [21] used tweets related to COVID-19 in the Los Angeles area, along with policy announcements and disease spread statistics, to construct a KG representing the real-world spread of COVID-19 in the Los Angeles area. The sentiment of each tweet was calculated using a rule-based method, and topic modeling was used to extract popular keywords from tweets.

The novelty of our constructed KG lies in domain specificity, the inclusion of patient-focused information from different sources, and the application of combining different information extraction methods. Most other medical KGs focus on the entire medical field and will therefore lose granularity on specific medical topics. We created a KG for NDDs involving input from patients and caretakers affected by NDDs, along with medical professionals who specialize in NDDs. This resulted in a KG containing more extensive knowledge about NDDs than a general medical KG. Input from patients and caretakers allowed us to include resources related to core knowledge, financial help, education, and services. This is in contrast to most other medical KGs, which only focus on extracting medical knowledge from the literature. In addition to the NER pipeline to detect standard terminologies used by medical professionals, we used topic modeling to capture resource-specific keywords. Using both NER and topic modeling allowed us to better annotate the resources. Furthermore, document classification was applied to categorize and label the resources into core knowledge, financial help, education, and services. Representing the extracted knowledge along with the resources in a KG leads to a centralized hub that combines resources from different areas of need around NDDs to maximize knowledge capture.

## Methods

### Overview

**Figure 1:**
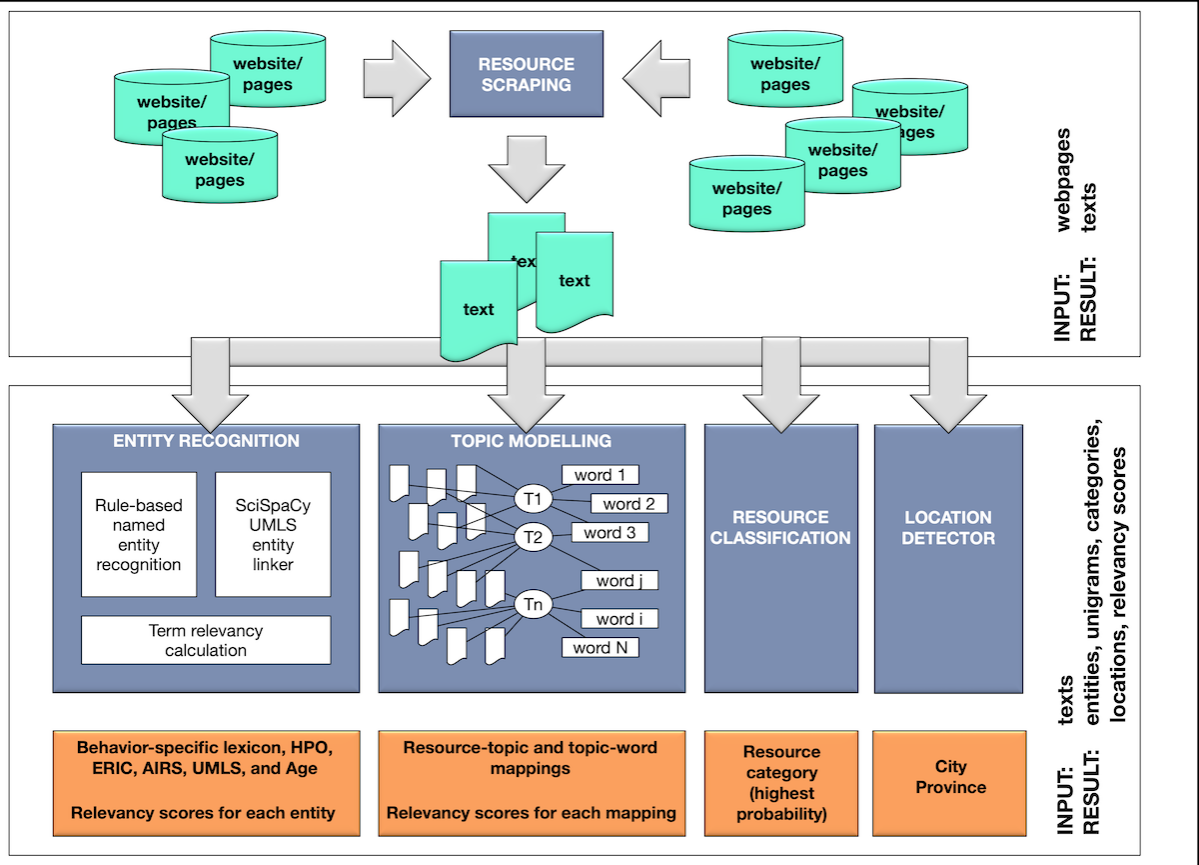
Annotation process of the resource website/pages. AIRS: Alliance of Information and Referral System; ERIC: Education Resources Information Center; HPO: Human Phenotype Ontology; UMLS: Unified Medical Language System.

Constructing a KG requires data and methods to represent these data in a format suitable to be a part of the KG. The following sections outline how data were collected and the methodology used to process the data for graph construction purposes. The proposed and applied methodology is illustrated in Figure 1. Data processing methods used to analyze text from websites included NER, topic modeling, document classification, and location detection.

### Data Collection

Two sources were used to construct the NDD corpus of text required for the KG. The first source included individuals with lived experiences who were part of the family advisory board or were recruited through advertisements for the project and community support groups focused on NDDs (AIDE Canada [22] and the Alberta Children’s Hospital NDD Care Coordination Project [23]). These individuals/parents were asked to provide links to websites relating to NDDs in such categories as core knowledge, education, services, and funding. The corpus created from these sources is referred to as the *NDD Caregiver subset*.

The second source of relevant web pages used for scraping was the Inform Alberta website [24]. This is referred to as the *Inform Alberta subset*. Finally, the combined list of web pages from both sources and some relevant pages added by the authors were scraped using the Python Scrapy library. For home pages, the entire site was scraped, while for specific/single web pages, only those pages were scraped.

As a result, the obtained corpus consisted of 200,000 web pages, with 80,000 pages from the *NDD Caregiver subset* and 120,000 pages from the *Inform Alberta subset*. HTML text was extracted for each page and cleaned by removing boilerplate text using the Python BoilerPy3 library. The collection of cleaned HTML text from the web pages formed the corpus of documents used for the construction of the KG-based repository of NDD resources.

### NER Approach

The list of NDD resources contained a mixture of website, home page, and web page URLs. To perform web page–level indexing, when a given URL referred to a home page/website, the Scrapy framework was used to scrape all the web pages of that website. Repetitive URLs were removed from the final list of all the web pages. Many web pages contained the same HTML boilerplate, such as headers, navigation bars, and footers. The Boilerpy3 Python library was used to remove this boilerplate HTML.

#### Entity Vocabulary

The data set contained various web pages related to services, education, financial help, and core health knowledge within the NDD field. Different standard terminologies, including those related to the Unified Medical Language System (UMLS), Human Phenotype Ontology (HPO), Education Resources Information Center (ERIC) thesaurus, and Alliance of Information and Referral Systems (AIRS) taxonomy, were extracted from the pages. In the constructed graph, they were used to annotate the web page URLs.

The UMLS is a collection containing over 4 million concepts from over 100 controlled vocabularies including but not limited to ICD-10 (International Classification of Diseases, Tenth Revision), MeSH (Medical Subject Headings), and SNOMED-CT (Systematized Nomenclature of Medicine-Clinical Terms) [25]. It covers all medical and related entities. The HPO provides a standardized vocabulary of phenotypic abnormalities encountered in human diseases. It currently contains over 13,000 terms [26]. The ERIC thesaurus is a list of topics in education and comprises about 11,818 terms, including 4552 unique terms called descriptors and 7133 synonyms of descriptors [27]. The AIRS taxonomy is the North American standard for indexing and accessing human service resource databases [28]. The taxonomy is a hierarchical system containing more than 9000 terms covering the complete range of human services.

As some web pages were more specific to a particular age as well as location, a list of age terms and all Canadian cities and provinces was used to index web pages. As behavioral issues are common in individuals with NDDs, with expert feedback, the following 10 categories of challenging behaviors were considered: sleep issues, sensory issues, hyperactivity, inattention, repetitive behavior, speech and language development, adaptive behavior, cognitive development, social skills, and behavioral concerns. For each category, we collected commonly used phrases or synonyms with the help of the parent advisory group, as well as performed a manual search on the UMLS interface [29].

#### NER Process

NER is a subtask of natural language understanding used to detect named entities that refer to specific objects. The named entities we used were domain-specific terms such as medical terms, educational terms, services, challenging behaviors, age, and location. All controlled vocabulary terms were given an entity label the same as their source vocabularies (ie, HPO, ERIC, AIRS, age, and location). Similarly, all challenging behavior phrases or vocabularies were labeled with their respective categories. They were lemmatized using the NLTK library [30]. A single pattern file was created as an input into SpaCy’s rule-based entity recognition component called EntityRuler. A pattern file is a dictionary with 2 keys: a “label” specifying the label to be assigned to the entity if the pattern is matched, and a “pattern” indicating the phrase to be matched. Web page text was preprocessed by removing stop words and lemmatizing the text, and was passed to EntityRuler to annotate the text. The UMLS Entity Linker from an open-source framework SciSpaCy [31] was used to extract UMLS entities from the text, and only the respective canonical concepts of UMLS entities were considered for further analysis.

#### Entity Relevance Calculation

Indexing the web pages with the existence or nonexistence of an entity does not provide information if a document is more relevant to a given entity. A document that mentions a given entity more often than other documents could be considered more relevant to this entity. Depending upon the number of occurrences of an entity, a weight is assigned to each entity, which is called an *entity relevance weight*. The weight provides information on how relevant an entity is to a document. However, using the term (entity) frequency alone will favor common words as well as long documents [32].

It is essential to normalize the term (entity) frequency to incorporate such factors as high term frequency and document length. This is especially so in the case of HTML documents because of keyword stuffing, a process where website owners deliberately add specific keywords to their site in order to improve its search engine ranking. We used logarithmic term frequency as a way to de-emphasize high-frequency terms and adjust within-document term frequency.

For normalization, the *pivoted unique normalization* method was used, which considers the document length as a factor. The principle of the pivoted normalization is as follows: the higher the value of the normalization factor for a document, the lower the chances of its retrieval. Therefore, to boost the chances of retrieving documents of a certain length, the normalization factor for those documents should be lowered. Singal et al [32,33] suggested considering the average document length in a corpus as a reference point, called *pivot*, and using a parameter called *slope* to penalize longer documents and give higher weight to shorter documents. Normalized term relevancy weight is defined as follows:

relevance = 1 + log(tf) / (1-slope) × pivot + (slope × dl) **(1)**

where *tf* is the term frequency in the document, and *slope* is set to 0.2 as suggested in the work by Singal et al. The value of *pivot* is set to the average number of distinct named entities per document in the entire collection, and *dl* is the length of the documents referred to by the unique number of entities in the documents. Documents with *dl = pivot* are not penalized as the normalization factor is simply equal to the *pivot.* For *dl > pivot*, documents are penalized and have lower chances of retrieval, while for *dl < pivot*, documents are rewarded with a smaller normalization factor.

### Topic Modeling

Topic modeling using latent Dirichlet allocation (LDA) was used to extract similar topics across the corpus for inclusion in the KG. A novel form of topic modeling, referred to as hierarchical topic modeling (HTM), was used to extract more specific topics from the corpus. Topic modeling was performed separately on the *NDD Caregiver subset* and on the *Inform Alberta subset* of the corpus due to computational constraints. Unigram topics were extracted.

#### Data Preparation

Each web page (document) in a subset of the corpus was preprocessed before being transformed into a count vector for modeling with LDA. The first step was to remove all punctuations from the document, followed by changing all words to lowercase. Next, the document was tokenized and lemmatized. Finally, a stop list was used to remove unwanted words from the document. The stop list used here was the default English stop list from NLTK augmented with some words added by the authors through iterative testing and analysis of the topic modeling outputs. Finally, preprocessed documents were transformed into a count vector for LDA.

#### Process Description

The HTM algorithm initially performed LDA on the corpus subset and reperformed LDA on topics containing several documents greater than a chosen threshold. Then, the process was repeated until each topic included less than the threshold number of documents, or no more progress was made. It resulted in more specific topic words than running LDA once over the whole corpus, as found by a subjective analysis comparing the outputs of both methods. The LDA algorithm from the Python scikit-learn library was used with the following hyperparameters: maximum iterations of 10, online learning algorithm, learning decay of 0.7, batch size of 128, and maximum features of 50,000.

For the *NDD Caregiver subset* of the corpus, the initial LDA was set to have 200 topics, and for the *Inform Alberta subset*, it was set to have 300 topics. These numbers were chosen to be in proportion to the number of documents in each corpus subset. The threshold for hierarchy termination was set to 300 documents for both corpus subsets. Only the lowest level of the topic hierarchy was used for the KG construction, as these topics seemed to be the most relevant following a subjective analysis.

#### Topic Relevance Calculation

It is essential to have information about the “strength” of connections among identified topics, documents (web pages), and unigrams, that is, words identified by LDA as describing each topic and indirectly representing documents associated with a given topic. In the case of LDA, such information was extracted from the LDA algorithm.

### Document Classification

There were 5 categories of web pages in the corpus: “financial help,” “education,” “services,” “core knowledge/health,” and “other.” To automatically label each web page, a few classification models were investigated. To construct models, a subset of the corpus was hand labeled as belonging to one or more of the 5 categories. This is a multilabel classification task, as documents (web pages) can belong to more than one category.

The hand-labeled data consisted of 2158 documents, with 116 labeled as “financial help,” 420 as “education,” 1419 as “services,” 1024 as “core knowledge/health,” and 143 as “other.” This data set was highly unbalanced. The data set was split into training, validation, and testing sets, with 80% of the data used for training, 10% for validation, and 10% for testing. The data were split equally along categories where possible.

We tested 3 groups of models for classifying these documents: (1) multilabel k-nearest neighbors, (2) 5 single-label transformers, and (3) a multilabel transformer. Among these models, we ultimately chose the multilabel transformer, as it achieved the highest macro F1 score on a held-out test set. The multilabel transformer was a 6-layer version of MiniLM-v2 fine-tuned on the prepared training data set. The pretrained model found on the HuggingFace website named nreimers/MiniLM-L6-H384-uncased was used; it is the same model as the *all-MiniLM-L6-v2* from *Sentence-BERT* [34]. A dropout layer with a dropout probability of 0.3 and a final sigmoid activation layer with 5 outputs were added to the base model as a multilabel classification head.

Training hyperparameters for this model were as follows. The loss function used for training was the binary cross entropy loss that was optimized using the AdamW optimizer. The optimizer learning rate was 3×10^−4^, with a linear warmup to this value and cosine decay to one-tenth of this value during training. The other optimizer hyperparameters were β_1_=0.9, β_2_=0.95, ϵ=1×10^−8^, and weight decay=0.01. The batch size was 64, and all gradients were clipped to a norm of 1.0 to mitigate gradient explosion.

The model was fine-tuned for 20 epochs. This is higher than the 2 to 3 epochs used in the original BERT paper [35], but we mitigated possible overfitting by increasing the dropout probability and evaluating model performance on a held-out validation set after each epoch. The model with the best performance on the validation set was chosen as the final model. The model outputs 5 probabilities between 0.0 and 1.0. A threshold value was chosen, where values above this threshold were considered members of the corresponding class. Finally, a more fine-tuned macro F1 score was calculated on the validation data set for threshold values from 0.0 to 1.0 in intervals of 0.1.

The best version of the multilabel transformer model, determined based on the validation set, achieved a macro F1 score of 0.504 and an accuracy of 84.1% on the testing set. For reference, the training set macro F1 score was 0.804, with an accuracy of 93.8%. Details of selected model performance can be found in Multimedia Appendix 1.

### Location Detection

Using regular expressions, link text was matched to scrape specific pages such as “contact us,” “our locations,” and “locate us.” Then, Canadian/US postal codes were matched using regular expressions and queried using the Google Maps application programming interface to get the city and province for a given postal code. Named entities were detected, along with cities and provinces. To get the final annotations, results from both modules were combined. As it was challenging to remove false-positive location entities due to manual annotation requirements, each city/province was given a weight equal to the proportion of entities that refer to the city/province. This way, for a given city/province, resources could be ranked based upon the score.

### Ethical Considerations

This project was approved by the research ethics board at the University of Alberta (study ID: Pro00081113). All the web pages that were analyzed are in the public domain.

## Results

### Overview

The presented methodology of processing resources (ie, web pages) provides a collection of items (ie, entities, unigrams, age ranges, locations, and web page categories) and challenging behaviors used to annotate the web pages. The integration of this information was done using a KG (KG-based repository of NDD resources). The web pages and items mentioned above were nodes, while the relevance between them was represented as edges labeled with a relevancy strength.

### KG Schema

A KG-based repository of NDD resources, as any KG, is a network of entities connected through relations. Each piece of knowledge in a KG is represented as a triple, with 2 entities connected through a relation. These triples are in the form of *(subject, relation, object)*. For example, to represent the piece of information that Edmonton is the capital of Alberta in a graph, the following triple is used: *(Edmonton, capital of, Alberta)*.

To effectively use a KG, names representing types of KG nodes and relations between the nodes must be established. This set, called *vocabulary*, is one of the essential aspects of constructing a KG. The vocabulary is often called the KG schema.

**Figure 2:**
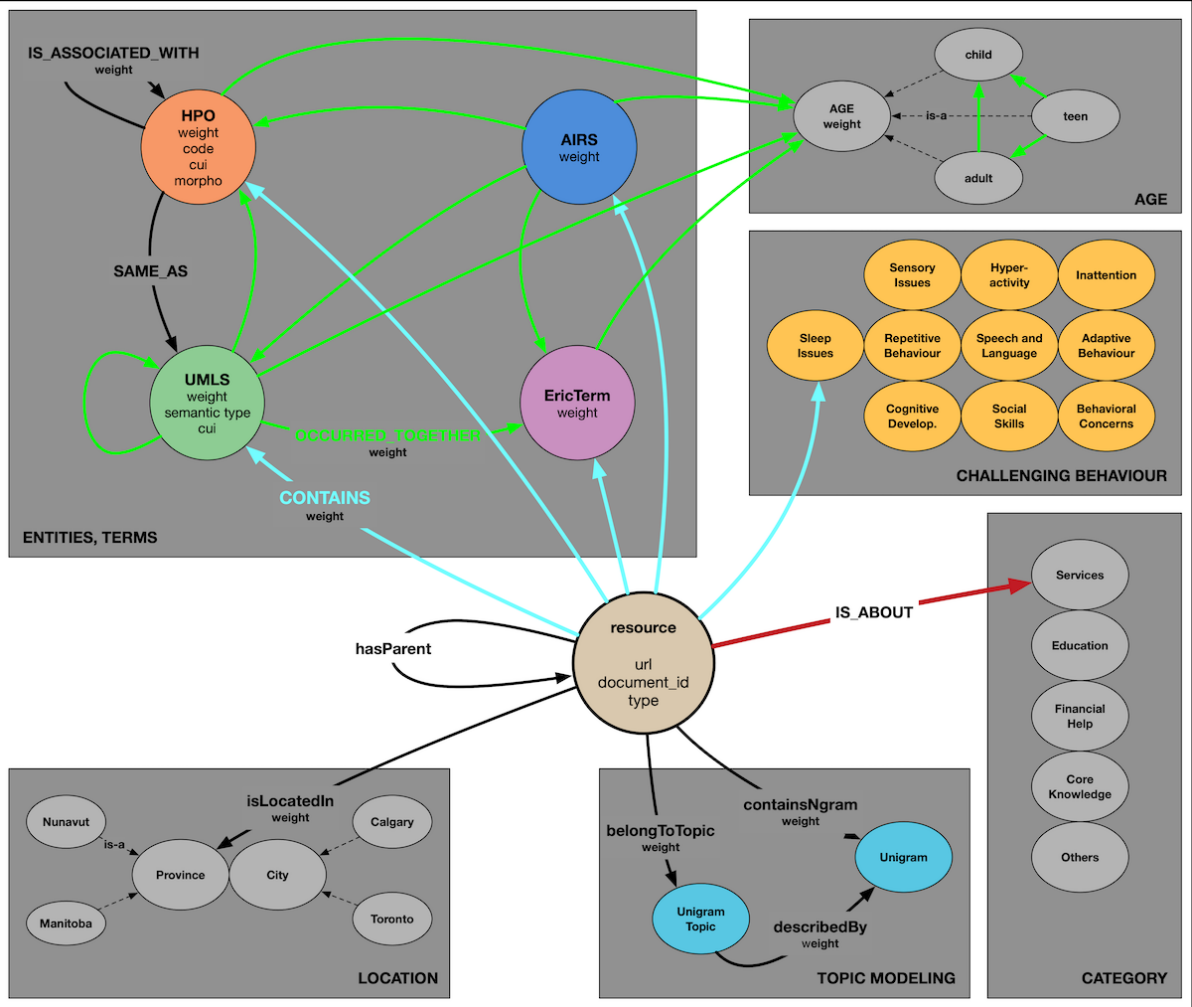
Knowledge graph schema. Links of the same color represent the same relations; dashed links represent the relation “is-a.” AIRS: Alliance of Information and Referral System; ERIC: Education Resources Information Center; HPO: Human Phenotype Ontology; UMLS: Unified Medical Language System.

The KG-based repository of NDD resources schema is shown in Figure 2. Names of node types are represented by circles, differing in color by node type. Some of them are labeled with extra information, shown in the text inside the node. Links between the nodes represent relations between entities. Some relations are labeled with extra information, shown in the text on the relation arrow. Each node type and relation type are outlined in the following sections. All collected data were represented as triples and fed into Neo4j to construct the graph automatically.

#### Entities as Nodes

There were a total of 11 node types in the KG-based repository of NDD resources*.* The primary node type was *Resource*. It represented all the documents (web page URLs) in the corpus. Each of these resources was labeled with the associated URL from the corpus, the resource source (*NDD Caregiver* or *InformAlberta*), and the resource type (web page, video, or PDF). The document text was not saved in the KG for size reasons. Instead, an external file was kept with processed document text for each URL, and each URL could be accessed through the internet, assuming the web page was still active.

The other node types were linked by edges, either directly or indirectly, to a *resource* node. HPO, UMLS, EricTerm, AIRS, and challenging behavior nodes were extracted using NER methods. *EricTerm* nodes were from the ERIC database and were labeled by the canonical term for the recognized entity. *UMLS* nodes were from the UMLS database and were marked by the canonical term for the recognized entity, its semantic type, and concept unique identifiers. *HPO* nodes were from the HPO-DDD database and were labeled by the canonical term for the recognized entity, and unique human phenotype and concept unique identifiers. *AIRS* nodes were from the AIRS database and were labeled by the canonical term for the recognized entity.

Two types of nodes represented terms extracted from the topic modeling method. First, unique topic nodes were placed into the KG-based repository of NDD resources, and then, each resource (web page) was linked with the unigram topic. Further, each unique topic node was connected with the corresponding unigram terms that were represented as nodes in the KG-based repository of NDD resources.

The remaining node types were *province*, *city*, *age*, *category*, and *challenging behavior*. The *province* and *city* for each resource were extracted using methods outlined in the location detection method. The *age* associated with each resource was also extracted using similar methods. Possible subtypes for *age* are *child*, *teen*, and *adult*. The node type *category* had 5 subtypes: *services*, *education*, *financial help*, *core knowledge*, and *other*. Resources were linked to one of the subtypes after the classification method outlined in document classification was executed.

#### Relations and Weights

Nodes were connected by edges to one of eight types of relations. Web pages scraped from a parent website and represented as resource nodes were connected to the relation *hasParent*. Location nodes, for both cities and provinces, were connected to the relation *isLocatedIn*. *City* nodes were connected to their corresponding *province* nodes with the relation *inProvince*. NER-related nodes, *age* nodes, and *challenging behavior* nodes were connected to corresponding resource nodes with the relation *CONTAINS*. NER-related nodes were also connected to identically named entities from different databases with the *IS_ASSOCIATED_WITH* relation. NER-related nodes and *age* were connected to each other with the relation *OCCURRED_TOGETHER*.

Topic nodes were connected to corresponding resource nodes with the relation *belongsToTopic*. The relation *describedBy* was used to connect topic nodes to their contained topic word (unigram nodes). Finally, resources were directly connected to relevant unigrams with the *containsNgram* relation.

Relations were assigned a weight using various methods if applicable. The *inProvince*, *hasParent*, and *describedBy* relations had no weights. The relations *CONTAINS*, *IS_ ASSOCIATED_WITH*, and *isLocatedIn* were weighted using term relevancy as outlined in the NER method. The relation *OCCURRED_TOGETHER* was labeled with several co-occurrences of connected entities (nodes).

The relations *belongsToTopic*, *containsNgram*, and topic-related *describedBy* had weights calculated as the output of the LDA process. Weights for the *belongsToTopic* relation represented the degree of how strongly a resource belongs to each topic. Weights for the topic-related *describedBy* relation indicated how strongly each word in the topic vocabulary belongs to each topic. The *containsNgram* weights were obtained by matrix multiplication of the *belongsToTopic* and topic-related *describedBy* weight matrices.

### Constructed KG: Overview

The constructed KG-based repository of NDD resources contained 264,167 nodes. There were 185,986 resource nodes. For the NER-related approach, there were 2448 *AIRS* nodes, 11,617 *EricTerm* nodes, 4181 *HPO* nodes, and 41,599 *UMLS* nodes. For topic modeling, there were 14,373 unigram nodes and 2045 unigram topic nodes. In addition, there were 3 *age* nodes, 5 *category* nodes, 10 *challenging behavior* nodes, 1832 *city* nodes, and 68 *province*/*state* nodes. The graph contained a total of 22,621,522 relations.

**Figure 3:**
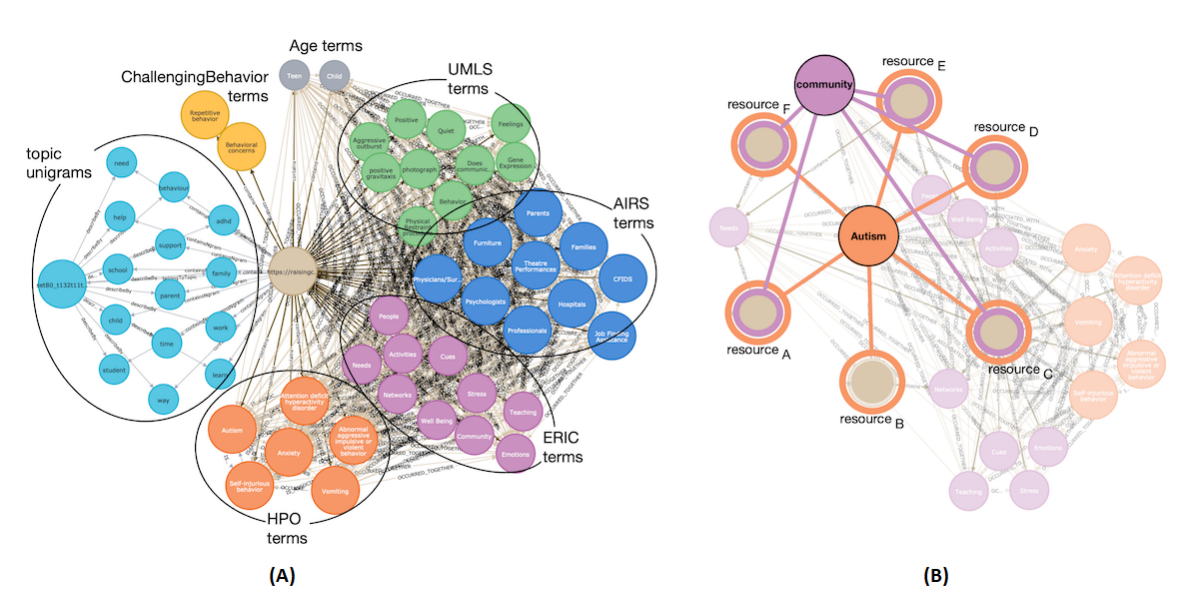
Example of an annotated resource: (A) most relevant annotating nodes; (B) n-to-n relations between resources and annotating nodes. AIRS: Alliance of Information and Referral System; ERIC: Education Resources Information Center; HPO: Human Phenotype Ontology; UMLS: Unified Medical Language System.

To illustrate interesting features of the graph, a single resource was extracted from the KG-based repository of NDD resources together with several annotated nodes (Figure 3A) [36]. The resource (light brown circle in the middle) is linked with a group of unigrams (blue circles on the left); 2 types of *challenging behavior* nodes (yellow circles); and *age* (gray), *UMLS* (green), *AIRS* (blue), *EricTerm* (violet), and *HPO* (orange) nodes.

These terms and unigrams define/describe the resource. There were n-to-n relations between resources and annotating nodes, meaning that a single annotating node was also linked with multiple resources. Such a scenario has been illustrated in Figure 3B. Two terms (HPO’s *autism* and EricTerm’s *community*) were connected to multiple resources.

**Figure 4:**
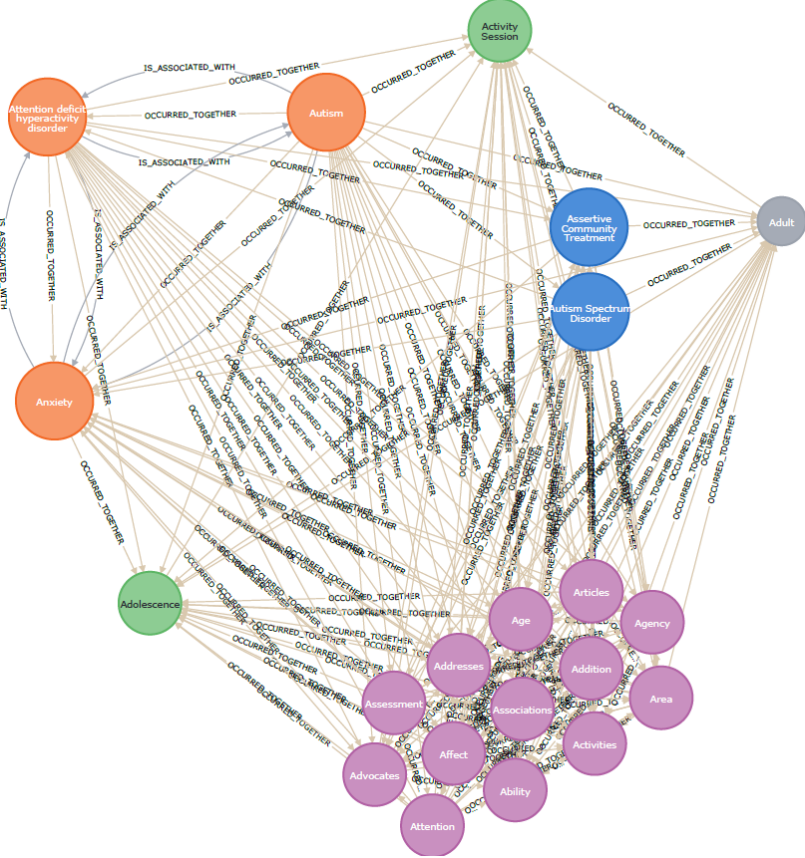
Example of the OCCURRED_TOGETHER and IS_ASSOCIATED_WITH connections between nodes. Entities from different sources are represented in different colors as follows: HPO, orange; AIRS, blue; UMLS, green; ERIC, purple; and age, grey.

Besides the relations between resources and annotating nodes, the graph contained multiple relations between annotating nodes. These were 2 types of relations (*OCCURRED_TOGETHER* and *IS_ASSOCIATED_WITH*). A fragment of the graph illustrating these relations is shown in Figure 4.

The existence of these many relations can create a highly interconnected representation of resources and their annotated nodes. However, it can introduce an issue if a user tries to identify the most relevant resources, as there would be no difference in relevance between nodes. Therefore, a degree of relevance was added to denote the most appropriate resources. The relevance represented the connection strength between a resource and an annotating node (entity).

Two types of relevance weights were used in the KG-based repository of NDD resources. One weight was linked with the relation *CONTAINS*. Its value was determined using the procedure presented in the entity relevance calculation method. The other weight was linked with the relation *OCCURRED_TOGETHER*. This weight is a measure of the co-occurrence of different annotating nodes.

**Figure 5:**
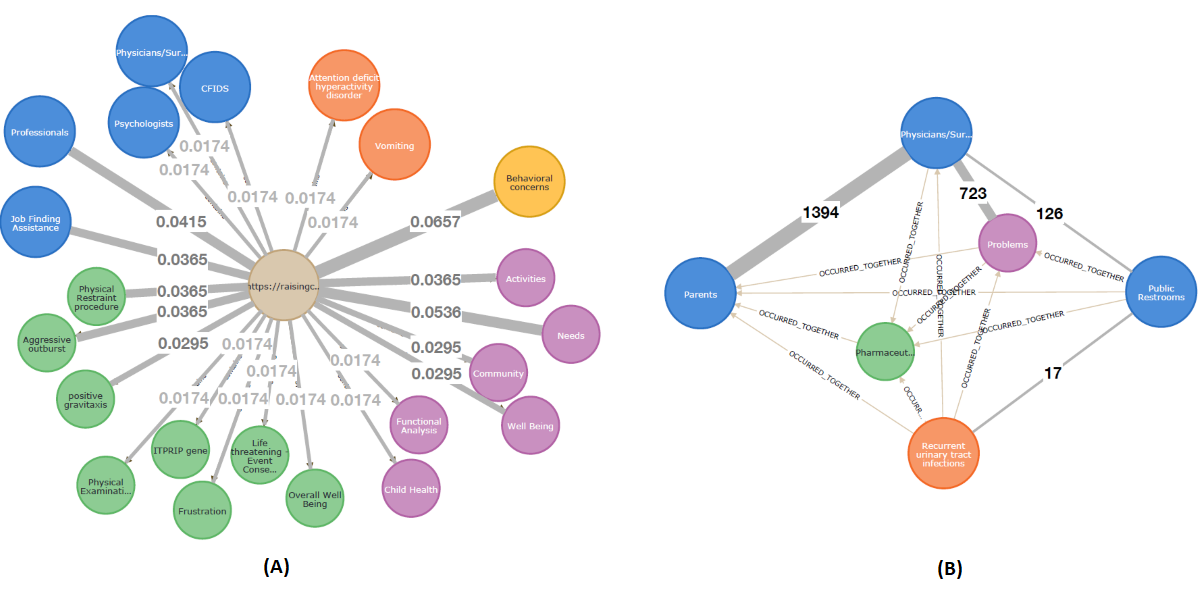
Example of the connection strength of (A) the relation CONTAINS between resources and annotating nodes and (B) the relation OCCURRED_TOGETHER between annotating nodes.

The first type of weight has been illustrated in Figure 5A. Although all connected nodes contribute to the description of a resource, their contributions are of different strengths. The second type of weight has been illustrated in Figure 5B. A snippet of the graph shows connections between annotating nodes (*HPO*, *AIRS*, and *EricTerm*). The weights were represented as integer numbers that indicated how often both terms co-occurred in the extracted text (ie, degrees to which the given nodes were “related to each other”).

### Use of the KG in a User Interface for Resource Retrieval

The KG-based repository of NDD resources can be used to identify the most relevant resources when a user provides some text input. A simple web-based interface has been developed to enable users to use the KG-based repository of NDD resources when they want to obtain a list of relevant resources. The interface allows the user to enter a text query containing several phrases representing their interest. Considering that end users can search with verbose queries, such as “my child hits other children at school,” to infer one of the 10 challenging behavior categories, we have also trained a text classification model that will classify the intent of the entered user text (Multimedia Appendix 2 [35,37-39]). Additionally, the richness of connections of the graph and the need to provide users with the best possible match to their text query led to the need for an aggregation operator that combined the weights (ie, the values of relevance between resources and annotating nodes) in a controlled way. This meant that if a user wanted to find the most relevant resource for a text query that satisfied multiple nodes, an OWA aggregation function was invoked. OWA combines all suitable weights for these nodes to rank the resources based upon their relevance (Multimedia Appendix 3 [13,40]).

The entered text is processed with the following steps:

1. Extract unigrams and entities (HPO, UMLS, ERIC, AIRS, and challenging behavior) using the developed natural language processing pipeline.
2. Classify user text as one of the 10 challenging behavior categories using the transfer learning–based text classification model. Then, add the detected category to the entities list obtained in step 1.
3. Query the KG-based repository of NDD resources to retrieve all resources that are connected to nodes representing entities and unigrams obtained in the above steps 1 and 2. For each retrieved resource, all annotating nodes are extracted together with the weights of the relations.
4. The weights are aggregated using OWA to determine the relevance of each retrieved resource.
5. A list of “sorted by relevance” resources is displayed to the user.

**Figure 6:**
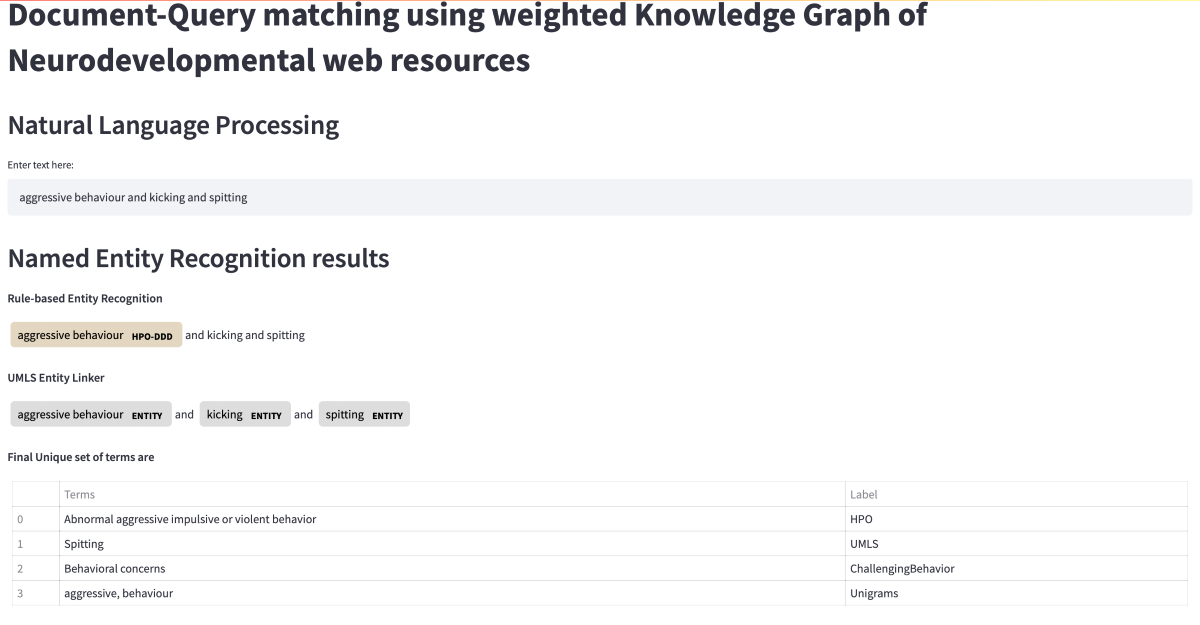
Question interface for query matching. The user enters the text “aggressive behavior and kicking and spitting” and obtains entities and unigrams.

**Figure 7:**
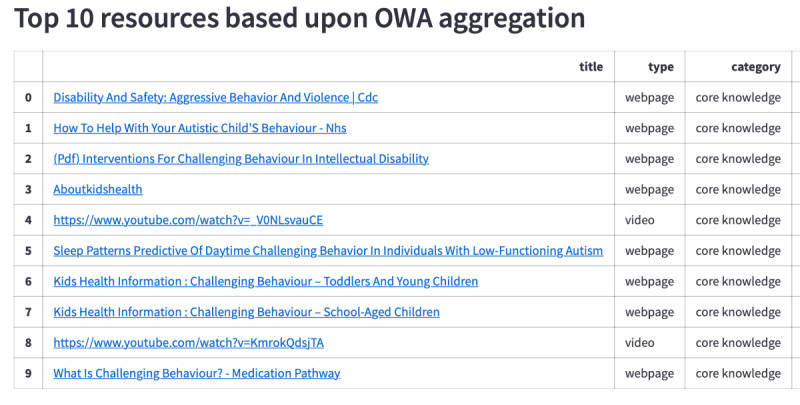
Question interface for the top 10 resources. List of the top 10 most relevant resources for the text “aggressive behavior and kicking and spitting.” OWA: ordered weighted averaging.

The text query, along with extracted entities and unigrams, is shown for a simple example in Figure 6. As can be seen, *HPO* and *UMLS* entities have been identified (“abnormal aggressive impulsive or violent behavior” and “spitting,” respectively). Additionally, *behavioral concerns* as a category of *challenging behavior* has been recognized. Two unigrams (“aggressive” and “behavior”) are extracted from the text. The resulting list of the most relevant sites has been determined and is shown in Figure 7. The list contains web pages and a video, all from the category *core knowledge*.

## Discussion

This paper describes the methodology for processing text extracted from web pages to generate a set of entities and terms used for annotating these web pages. Both web pages and entities/terms are the basis for constructing a knowledge base of resources. This base, built as a graph called the KG-based repository of NDD resources, is a highly interconnected network linking resources with annotating terms and entities. Furthermore, edges in the KG-based repository of NDD resources have weights representing relevance between resources and annotating terms/entities. Edge weights, aggregated using a specialized aggregation operator, are used to rank resources. The constructed KG-based repository of NDD resources is a repository of resources about NDDs that can be queried using textual phrases, with relevant results shown to the user using an interface.

Most of the prior work in building a medical KG has used scientific literature, such as PubMed and electronic medical records, and only specific types of entities, such as diseases, chemicals, and genes, have been considered [17,41,42]. Ernst et al [43] used patient-oriented online health portals to build a KG, indicating the importance of medical information spread across different sources. Shi et al [15] represented heterogeneous textual medical knowledge as a KG to use it further for semantic reasoning. Yu et al [44] constructed a KG for traditional Chinese medicine to integrate terms, documents, and databases in a base to facilitate sharing and use of traditional Chinese medicine health care knowledge.

To our knowledge, this is the first method to integrate credible online information from different areas of need around NDDs (ie, financial help, services, education, and core knowledge) into one base. Our developed natural language processing pipeline can be used to annotate resources from the abovementioned areas. Representing the extracted knowledge in a KG allows for finding connections among different resources on a scale that would be impossible for a single human. Many ontologies and scattered information at both professional and layperson levels exist on the internet, and our KG compiles all this information in a single place. Connecting all this information will open up many areas of improvement for the NDD field. These could include new research directions, new treatment opportunities, and the possibility of collaboration among services.

The methodology used to construct this KG is scalable and could be expanded to other medical domains besides NDDs. In creating more of these domain-specific medical KGs with the guidance of medical professionals, patients, and caretakers, we can provide information in a similar way to patients having other conditions. These specific KGs could even be connected at a higher level to slowly create a field-wide medical KG, which would be of great benefit in complex medical conditions where individuals present with multiple hyperspecialized domains. The bottom-up nature of the creation of this KG may result in a better product than the top-down field-wide medical KGs that currently exist.

While having more documents means more information is available, there is also a tradeoff between the number of documents and the KG query speed [45]. Some ways to overcome this include indexing the KG [46], and pruning irrelevant nodes and edges on the KG [47]. Another limitation of KG construction is that pivoted unique normalized logarithmic term frequency is used to calculate weights for edges labeled *CONTAINS*, which can affect the performance of the resource retrieval method when the size of the document is significantly greater than the average document length in the corpus. Pivoted unique normalization overpenalizes longer documents as shown in the original paper [48]. When the length of a document is much larger than the average document length in the corpus, a higher normalization factor could yield an almost zero relevance score for that document’s entities [48]. This limitation can be overcome by implementing a term relevance method, which considers not only the term frequency but also co-occurring terms (represented with the *OCCURRED_TOGETHER* relationship in the KG-based repository of NDD resources) [49]. As future work, the semantic search methods of the KG-based repository of NDD resources will be further studied to address the exact keyword matching issue in the resource retrieval system. Potential solutions include using query expansion techniques [50-52]. The application of OWA for identifying the most relevant list of resources opens another possibility of enhancing the user query interface. OWA is known for its ability to include linguistic quantifiers, such as SOME, MOST, ALL, and AT LEAST n, in the aggregation process. So far, we have only used MOST to aggregate query results, yet a user can control to what degree documents should satisfy different criteria using different quantifiers.

To further improve the user’s experience with the resource retrieval process of the KG-based repository of NDD resources, we aim to build a transparent interface that will enable path-based explanations in the KG-based repository of NDD resources to provide relevant background knowledge in a human-understandable format [53,54], using interpretable machine learning approaches. Explainable artificial intelligence is an emerging research field, which focuses on not only the performance of the models but also the interpretability of what factors led the model to make a particular decision. This promotes credibility and trust in the suggested results [55,56].

Although patients and caretakers were included in some of the vital steps of creating this KG, such as collecting resources and challenging behavior vocabulary, user feedback is an important step in the process of validating our created KG. We will design an evaluation strategy to validate the document retrieval system of the KG-based repository of NDD resources by collecting a gold standard relevance assessment from human judges. Douze et al [57] found that the relevance assessment from human judges depends upon their subjective needs. Therefore, we will collaborate with a group of parents of children with NDDs to create a gold standard test collection to evaluate the model and check if the document retrieval system of the KG-based repository of NDD resources satisfies their needs.

The need for helping families with NDDs could leverage the potential that online information has to offer (eg, to supplement gaps in the health/social support system). This need became more important than ever during the COVID-19 pandemic and will continue to gain importance in the future. Building an efficient repository of trusted web resources has proven to be challenging due to the lack of uniformly labeled resources. This challenge is not unique to NDDs and is seen across other medical fields as well. Such repositories of online resources should provide users with an intelligently generated ranking of resources based on a simple text query entered by the users. Experts and nonexperts can use the KG-based repository of NDD resources to improve the quality of life of people with NDDs. Future work includes enhancement of the user interface for resource retrieval, as well as mechanisms for continuous modification of the KG-based repository of NDD resources when new information is discovered or old information is found to be outdated.

## Appendix

Multimedia Appendix 1 Multilabel transformer model performance results.

Multimedia Appendix 2 Methodology of the language model–based challenging behavior detection.

Multimedia Appendix 3 Ordered weighted averaging–based resource relevance ranking algorithm.

## Abbreviations

AIRS: Alliance of Information and Referral Systems
ERIC: Education Resources Information Center
HPO: Human Phenotype Ontology
HTM: hierarchical topic modeling
KG: knowledge graph
LDA: latent Dirichlet allocation
NDD: neurodevelopmental disorder
NER: named entity recognition
OWA: ordered weighted averaging
UMLS: Unified Medical Language System
