# Supplementary material for: Leveraging Knowledge Graphs and Natural Language Processing for Automated Web Resource Labeling and Knowledge Mobilization in Neurodevelopmental Disorders: Development and Usability Study
Source: J Med Internet Res. 2023 Apr 17;25:e45268. doi: 10.2196/45268 (PMC10152329; doi:10.2196/45268)
Supplement: Multimedia Appendix 1 [file jmir_v25i1e45268_app1.docx]

**Multimedia Appendix 1.** Multilabel transformer model performance results.

| Label: | | Financial Help | Education | Services | Other | Core Knowledge / Health |
| --- | --- | --- | --- | --- | --- | --- |
| Train | Cases | 89 | 331 | 1131 | 111 | 815 |
|  | Precision | 0.745 | 0.769 | 0.920 | 0.756 | 0.860 |
|  | Recall | 0.787 | 0.764 | 0.984 | 0.532 | 0.977 |
|  | F1 | 0.765 | 0.767 | 0.951 | 0.624 | 0.914 |
| Test | Cases | 12 | 40 | 141 | 14 | 101 |
|  | Precision | 0.143 | 0.333 | 0.840 | 0.400 | 0.743 |
|  | Recall | 0.083 | 0.500 | 0.965 | 0.286 | 0.832 |
|  | F1 | 0.105 | 0.400 | 0.898 | 0.333 | 0.785 |

Number of cases, precision, recall, and f1 score for each class on held-out test set are shown.
