# Supplementary material for: Leveraging Knowledge Graphs and Natural Language Processing for Automated Web Resource Labeling and Knowledge Mobilization in Neurodevelopmental Disorders: Development and Usability Study
Source: J Med Internet Res. 2023 Apr 17;25:e45268. doi: 10.2196/45268 (PMC10152329; doi:10.2196/45268)
Supplement: Multimedia Appendix 2 [file jmir_v25i1e45268_app2.docx]

## **Multimedia Appendix 2.** Methodology of the language model–based challenging behavior detection.

## Introduction

Pre-trained language models with transformer architecture have led to significantly improved results compared to legacy methods [37]. The process of fine-tuning is done to adapt pre-trained language models to domain-specific tasks. Fine-tuning means further training of parameters with domain-specific data instead of generic information. The Bidirectional Encoder Representations from Transformers (BERT) model is an encoder-only transformer architecture [35]. BERT has bidirectional attention, and its pre-training is performed by masked language modeling (MLM), which randomly masks tokens, and the objective is to predict the masked token. Given BERT’s architecture, it can be fine-tuned with only one additional output layer.

## Dataset

The training dataset included 1,219 natural language descriptions of challenging behaviors, each annotated by the recruited parents group into one of the ten available categories. These ten categories are: Cognitive development, Sleep issues, Speech and language development, Sensory issues, Social skills, Hyperactivity, Behavioral concerns, Inattention, Adaptive behavior, and Repetitive behavior. A few examples of the text and their annotated categories can be seen in Table S1.

**Table S1**: Examples of text and their annotated categories.

| **Text** | **Category** |
| --- | --- |
| He just can’t deal with the work he needs to do in order to have help. | Adaptive behavior |
| Tendency to be over-sensitive to noise. | Sensory issues |
| He isn’t particularly interested in our weekday routines and mostly sleeps through them. | Sleep issues |

## Architecture

A pre-trained BERT language model with a fully-connected classification head was used to classify the natural language text into one of the ten annotated categories. The smaller version of the pre-trained BERT model, called the base model, was used to have faster inference time while still resulting in acceptable accuracy. The BERT-base model has almost 110 million parameters. The output of the pre-trained BERT model was passed through a Dropout layer with a probability of 0.1 to avoid overfitting to the training data [38]. The fully-connected layer has an output of ten features in order to be able to classify between the different classes.

## Experiments

The dataset was split randomly with a seed of 66 to training and validation sections with an 80/20 ratio. The pre-trained model used was BERT-base. The input text was truncated, and the maximum input size set to 128 tokens. The classifier model was trained AdamW optimizer [39] with a learning rate of 2e−5 and weight decay of 0.01 for ten epochs. The code was written in PyTorch. Fine-tuning was done using an NVIDIA Titan RTX GPU.

The fine-tuned model resulted in an accuracy of 85.7% on the validation dataset. Among the different classes, the model was the most confused when text regarding repetitive behavior was given, and it incorrectly predicted behavioral concerns. The full confusion matrix can be found in Figure S1.

## Inference

The final trained model has a disk space size of 433 MB. The model is hosted on a remote server and is served using an API. Remote hosting of the classification model results in leaner code for the chatbot. This modular architecture also results in an easy and independent update of the classification model without adversely affecting the rest of the chatbot architecture. The model is currently hosted on HuggingFace server 1 and, on average, has an inference time of less than 200 ms, which makes it a good fit for time-sensitive applications, such as our case of chatbots.


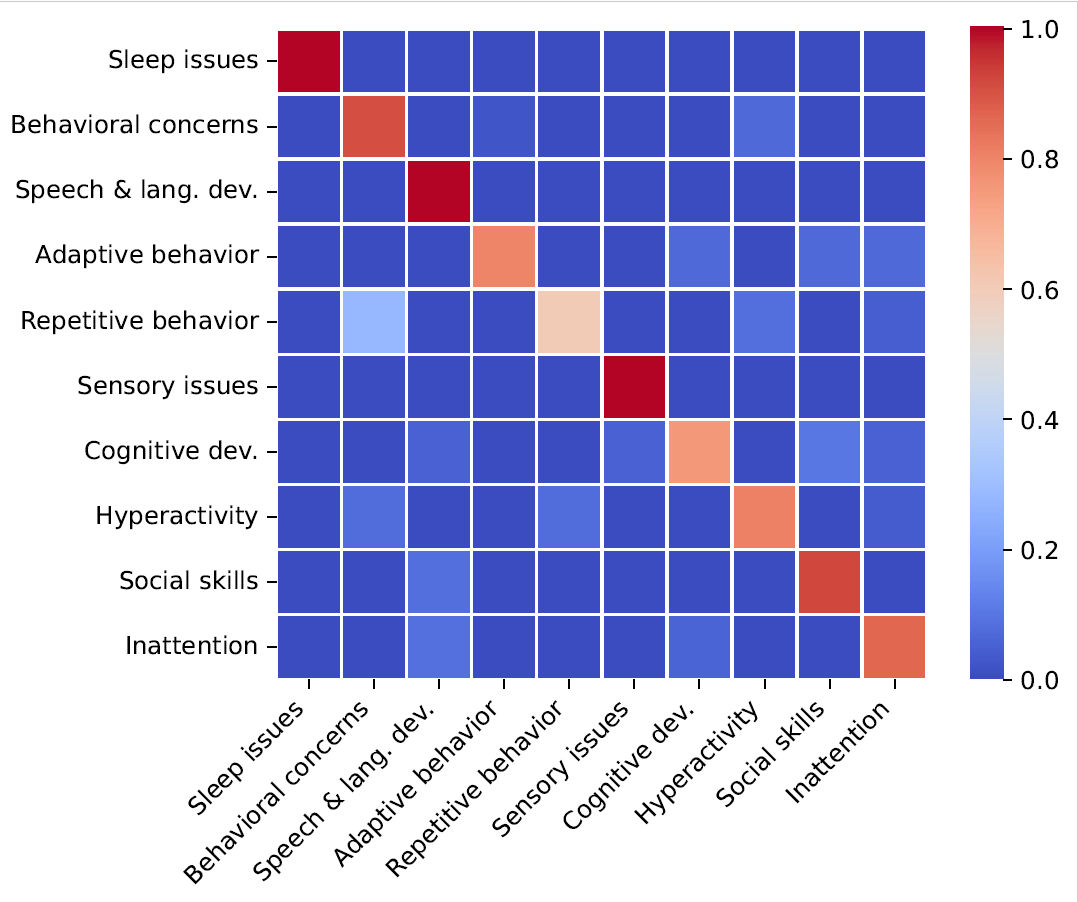


Figure S1: Normalized confusion matrix. Rows show the true labels, columns show the predicted labels.
