# Supplementary material for: Leveraging Knowledge Graphs and Natural Language Processing for Automated Web Resource Labeling and Knowledge Mobilization in Neurodevelopmental Disorders: Development and Usability Study
Source: J Med Internet Res. 2023 Apr 17;25:e45268. doi: 10.2196/45268 (PMC10152329; doi:10.2196/45268)
Supplement: Multimedia Appendix 3 [file jmir_v25i1e45268_app3.docx]

# **Multimedia Appendix 3.** Ordered weighted averaging–based resource relevance ranking algorithm.

## Introduction to Ordered Weighted Averaging

We use the Ordered Weighted Averaging (OWA) operator [13], which is a weighted sum of ordered pieces of information. The OWA operator, defined on the unit interval *𝐼* and having dimension *𝑛*, is a mapping

*𝑂𝑊 𝐴* ∶ *𝐼 ^𝑛^* _⟶_ *𝐼* such that

*OWA (a_1_, a_2_, …, a_n_) = ∑_j = 1 to n_ w_j_ * b_j_* (1)

Where b_j_ is the j^th^ largest of the a_i_’s. W = {w_1_, w_2_, …, w_n_} is a weighting vector such that 0 ≤ w_j_ ≤ 1 and ∑_j = 1 to n_ w_j_ = 1

To obtain a weighting vector W associated with an OWA, a family of RIM quantifiers Q has been introduced. A fuzzy subset Q represents a RIM quantifier if:

1) Q(0) = 0;

2) Q(1) = 1;

3) If r_1_ > r_2_ then Q(r_1_) > Q(r_2_)

Assuming a RIM quantifier Q, the weighting vector W can be determined such that for j = 1 to n:

W_j_ = Q( j/n) - Q ((j-1)/n) (2)

A function Q can be of different form and be associated with different linguistic quantifiers, such as *for all*, *mean*, *most*, or *as many as possible* [40]. In the paper, the quantifier *most* is used, which leads to the following form of Q:

Q (r) = 0 if 0 ≤ r ≤ α

Q (r) = r-α / β-α if α ≤ r ≤ β

Q (r) = 1 if β ≤ r ≤ 1 (3)

where α = 0.3 and β = 0.8.

## Resource Ranking Process

The process for identifying the list of most relevant resources is outlined in Algorithm 1. The algorithm takes *NDD-KG* and the user’s text query as its input. The phrase is processed, and sets of entities and unigrams are obtained (lines 11 and 12). Based on both sets, a *Neo4j* query is created, and a set of resources is returned (line 14).

The weights associated with the CONTAIN relations are used to determine relevance. The weights of all CONTAIN relations are extracted (lines 16 to 19). Similarly, the weights of edges connecting resources and unigrams are retrieved.

The retrieved weights - *Weight^E^_i_* and *Weight^UG^_i_* for a resource *Res_i_* are aggregated individually using OWA, and then the results are multiplied (line 24). The obtained value – *significance_i_* – is used to determine a ranking of all resources that satisfy the user’s text query.

| Algorithm 1. Resource Ranking Algorithm |
| --- |
| 1: Input:  2: NDD-KG  3: UInput *⊳* User_Input_Phrase  4: Output:  5: RRL *⊳* Ranked_Resource_List  6: Initialization:  7: set: UEntity = {} *⊳* Entities extracted from UInput  8: set: UuniGram = {} *⊳* Unigrams extracted from UInput  9: set: SRes = {} *⊳* Selected Resources  10: list: RRL = []  11: UEntity ← entityExtraction(UInput)  12: UUniGram ← unigramExtraction(UInput)  13: Neo4j_query ← queryConstruction(UEntity U UUniGram)  14: SRes ← execute(Neo4j_query)  15: for each Res_i_ from SRes do  16: for each entity_j_ from UEntity do  17: weight^E^_i, j_ ← getWeight(Res_i_ – contains – entity_j_)  18: Weight^E^_i_ ← weight^E^_i, j_  19: end_for  20: for each unigram_j_ from UuniGram do  21: weight^UG^_i, j_ ← getWeight(Res_i_ – contains – unigram_j_)  22: Weight^UG^_i_ ← weight^UG^_i, j_  23: end_for  24: significance_i_ = OWA(Weight^E^_i_) * OWA(Weight^UG^_i_)  25: ResList_significance ← (Res_i_, significance_i_)  26: end_for  27: RRL ← ranking(ResList_significance)  28: return |

#### 
